# Non-inferior comparative study comparing one or two day antimicrobial prophylaxis after clean orthopaedic surgery (NOCOTA study): a study protocol for a cluster pseudo-randomized controlled trial comparing duration of antibiotic prophylaxis

**DOI:** 10.1186/s12891-019-2879-3

**Published:** 2019-11-13

**Authors:** Kosei Nagata, Koji Yamada, Tomohiro Shinozaki, Tsuyoshi Miyazaki, Fumiaki Tokimura, Hiroyuki Oka, Yasuhito Tajiri, Sakae Tanaka, Hiroshi Okazaki

**Affiliations:** 1 Department of Orthopaedic Surgery and Spinal Surgery, The University of Tokyo Hospital, Tokyo, Japan; 2 Department of Orthopedic Surgery, Japan Organization of Occupational Health and Safety Kanto Rosai Hospital, 1-1 Kizukisumiyoshi-cho, Nakahara-ku, Kawasaki City, Kanagawa Prefecture 211-8510 Japan; 3 Department of Information and Computer Technology, Faculty of Engineering, Tokyo University of Science, Tokyo, Japan; 4 Department of Orthopedic Surgery, Tokyo Metropolitan Geriatric Hospital and institute of Gerontology, Tokyo, Japan; 5 Department of Medical Research and Management for Musculoskeletal Pain, 22nd Century Medical and Research Center, Faculty of Medicine, The University of Tokyo, Bunkyo-ku, Tokyo, Japan; 6 Department of Orthopedic Surgery, Tokyo Metropolitan Hiroo Hospital, Tokyo, Japan

## Abstract

**Background:**

Antimicrobial prophylaxis (AMP) is one of the most important measures for preventing surgical site infections (SSIs); however, controversies remain regarding its adequate duration. Although the World Health Organization and the Center for Disease Control and Prevention do not recommend additional AMP after closure, the American Society of Health-System Pharmacists and the Musculoskeletal Infection Society permit the use of postoperative AMP, but recommend discontinuation within 24 h. Similarly, the Japanese Society of Chemotherapy and the Japan Society for Surgical Infection also permit AMP within 24–48 h after various orthopaedic procedures. In these guidelines, recommendations regarding AMP duration were weak due to a relative lack of evidence, and currently, there is no high-quality evidence comparing AMP use within 24 h versus 24–48 h regarding orthopaedic procedures. Urinary tract infection (UTI) and respiratory tract infection (RTI) are also important health care-associated infections (HAIs) faced after surgery. Although AMP duration may affect these HAIs, its effects have not been well evaluated.

**Methods:**

We have organized a multicenter, prospective, cluster pseudo-randomized controlled trial to examine the non-inferiority of shorter AMP duration (within 24 h) against longer duration (24–48 h) in preventing postoperative HAIs. Participating facilities will be divided into two groups. In Group 24, AMP will be discontinued within 24 h after surgery. In Group 48, AMP will be discontinued within 24–48 h after surgery. The group allocation will be switched every 2 months until the targeted recruitment (500 participants per group) is met.

The primary outcome will be the cumulative incidence of all HAIs (SSI, UTI, RTI, and other infectious diseases), which require antibiotic therapies within 30 days after surgery. In addition to mortality and cardiovascular events, prolonged hospitalization (> 30 days) and the rate of antibiotic resistance rate of SSI pathogens will also be evaluated. Outcomes will be evaluated within 30–180 days after surgery in person by the surgeon, by mail, or by telephone survey. Data will be analyzed by a statistician not engaged in data collection.

**Discussion:**

This study may provide valuable information for developing future recommendations for adequate AMP duration after clean orthopaedic surgery.

**Trial registration:**

UMIN000030929, registered January 22, 2018.

## Background

Antimicrobial prophylaxis (AMP) is one of the most important measures for preventing surgical site infections (SSIs) in various orthopaedic procedures [1, 2], (http://www.chemotherapy.or.jp/guideline/jyutsugo_shiyou_jissen.pdf), [3]. Generally. AMP is not encouraged for long durations postoperatively, but adequate AMP duration remains controversial in clean orthopaedic surgery. The American Society of Health-System Pharmacists (ASHP) [4] and the Musculoskeletal Infection Society (MSIS) [5] permit postoperative AMP administration, but recommend discontinuation within 24 h after surgery. On the contrary, the recent guidelines from the World Health Organization (WHO) [2] and the Center for Disease Control and Prevention (CDC) [3] recommend AMP administration only for the intraoperative period, with no additional doses after the closure. Theoretically, shorter duration may be superior for not contributing to antibiotic resistance; however, no high-quality evidence in the field of orthopaedic surgery has demonstrated reduction in SSI rate or resistant pathogens by restricting AMP duration to the intraoperative period only [1], (http://www.chemotherapy.or.jp/guideline/jyutsugo_shiyou_jissen.pdf).

**Fig. 1:**
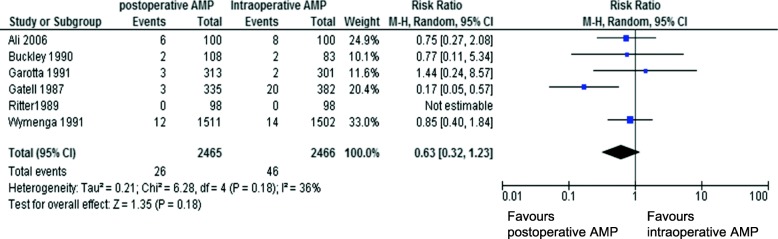
Postoperative vs. intraoperative antimicrobial prophylaxis of orthopedic surgery in CDC guideline, outcome SSI

**Fig. 2:**
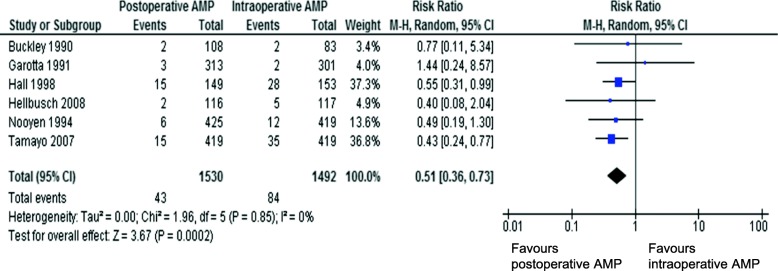
Postoperative vs. intraoperative antimicrobial prophylaxis of clean orthopedic, cardiovascular and thoracic surgeries in WHO guideline, outcome SSI

There are several limitations of the WHO and CDC guidelines regarding the generalization of orthopaedic procedures. First, although the WHO developed guidelines following GRADE (Grading of Recommendations Assessment, Development and Evaluation) methods based on randomized control trials (RCTs) [2, 6], only a few studies on orthopaedic surgeries were cited and these studies mostly had small sample size [7–9]. Second, although CDC guidelines cited 21 RCTs (*N* = 14,285) to conclude that there was no significant difference in SSI rates between intraoperative AMP and those discontinued within 24 h after surgery (*P* = 0.15) [3], there were only six orthopaedic-related studies (four on fracture surgery, and two on arthroplasty) [7, 8, 10–13]. The pooled risk ratio of these six studies showed no difference in the SSI rate [7, 8, 11–13], but there was a trend towards favoring postoperative AMP (pooled risk ratio 0.63 (95% CI 0.32–1.23, *P* = 0.18), with one study reporting higher SSI rate with intraoperative AMP (Fig. 1) [3]. Moreover, in WHO guideline, when the data were limited to clean orthopaedic, cardiovascular and thoracic surgeries, the pooled risk ratio of the quoted literatures was 0.51 (95%CI 0.36–0.73), significantly favoring postoperative AMP (Fig. 2). Among these studies, 2 studies showed significant SSI reduction with postoperative AMP, while no studies showed significant SSI reduction with intraoperative AMP [2]. From these available data of clean surgeries, the evidence for recommending intraoperative AMP seems weak and inadequate, and thus may not be appropriate for clean orthopaedic procedures.

The Japanese Society of Chemotherapy (JSC) and the Japan Society for Surgical Infection (JSSI) recently developed a practical guideline for AMP (http://www.chemotherapy.or.jp/guideline/jyutsugo_shiyou_jissen.pdf), making recommendations for each common procedure. In total, 11 orthopaedic procedures were selected. According to this guideline, there was insufficient evidence to support shorter AMP duration to prevent SSIs in any orthopaedic procedure [14]. In contrast, they cite evidence indicating that shorter duration may actually increase the risk of SSIs in several orthopaedic procedures (http://www.chemotherapy.or.jp/guideline/jyutsugo_shiyou_jissen.pdf), [15, 16]. From the subgroup analysis of the Cochrane review, a higher SSI risk was observed for a single-dose AMP compared with multiple-doses AMP in closed fracture surgery [16]. In another systematic review, postoperative AMP administration for > 24 h demonstrated lower SSI rate than shorter AMP duration for long-bone tumor surgery with endoprosthetic reconstruction [15]. For spinal surgery, only one RCT was conducted that compared intraoperative with prolonged AMP administration. In this study, the SSI rate was slightly higher in the intraoperative group, although there was no significant difference due to the small sample size [9].

SSIs with resistant pathogens following clean orthopaedic procedures are generally a disaster. Considering the potential risk of antibiotic resistance with longer AMP duration, the JSC/JSSI recommend that AMP be discontinued within 48 h after closure for spinal instrumentation surgeries [1, 9, 17] and arthroplasties [18, 19]. In contrast, the ASHP and MSIS recommend discontinuation of AMP within 24 h [4, 5] and the CDC and WHO recommend discontinuation even earlier [2, 3]. However, with the lack of high-volume comparative studies in the field of orthopaedic surgery, it remains unclear whether shorter AMP duration really decreases antibiotic resistance, or provides better outcomes after orthopaedic surgeries.

Health care–associated infections (HAIs), including postoperative urinary tract infection (UTI) and respiratory tract infection (RTI), may also become disastrous complications after orthopaedic surgeries. They are known to increase medical costs, prolong hospital stays, and increase mortality rates [20–22]. Theoretically, AMP may also be effective in preventing these HAIs [3, 6], but it is still unclear what clinical impact does AMP duration have on these complications following orthopaedic procedures. A large, well-designed comparative prospective study is needed to evaluate the association between AMP duration and HAIs after orthopaedic surgeries.

Considering the importance of all these infectious complications, we plan to conduct a comparative study evaluating the effectiveness of AMP duration on all HAIs, including SSIs, as a combined outcome. To the best of our knowledge, there has been no comparative trial in the field of orthopaedic surgery comparing the effectiveness of AMP duration for 24 h versus 24–48 h on these complications. In our previous study, AMP was continued for > 24 h in about half of our procedures [23]. Since none of the domestic SSI prevention guidelines recommend stopping AMP after closure [14], it is still not an established general practice to stop AMP without additional postoperative administration. Therefore, it was difficult to recruit participating hospitals to collaborate on a comparative study comparing intraoperative AMP alone with longer AMP duration. Moreover, although the JSC/JSSI recommend that AMP be discontinued within 48 h, there is not enough evidence to justify prolonged AMP of > 24 h following clean orthopaedic procedures. Accordingly, the objective of this study was to examine the effectiveness of AMP duration (24 h vs 24–48 h) on postoperative HAIs after clean orthopaedic surgeries.

## Methods

### Sample size

**Fig. 3:**
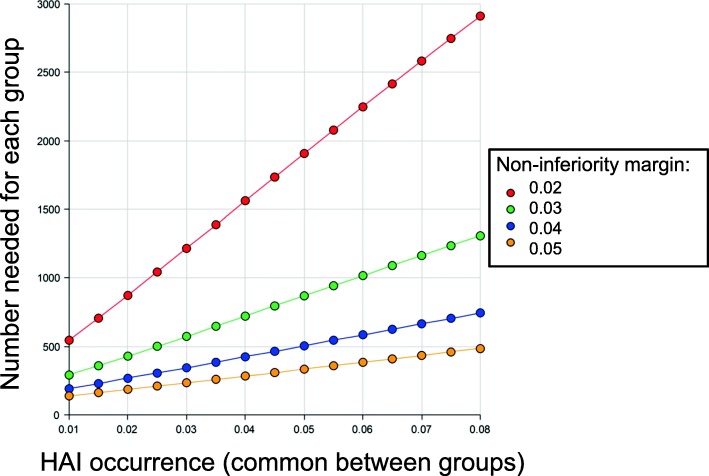
Line graph for sample size decision. The whole results of sample size calculation in the scenarios of the HAI risk ranging from 1 to 8% and the non-inferiority margin from 2 to 5%, assuming that the HAI occurrence probability is common between groups (1-sided Farrington–Manning test, with α = 2.5% and β = 20%)

This study design is a cluster pseudo-randomized, multicenter, non-inferiority comparative study (NOCOTA study). For this trial, the sample size was determined based on our previous research and feasibility. In a one-year survey of 2589 patients in the participating hospitals, 30-day HAIs were observed in 4.2% of patients after clean orthopaedic surgeries (on submission), and thus, we assumed that the risk of HAIs within 30 days after surgery would be 4% in both duration groups. The Farrington–Manning non-inferiority score test, with an alpha error of 0.05, required at least 400 participants per group to declare non-inferiority with a margin (i.e., an acceptable difference in HAI risks) of 4%, with a beta error of 0.2. To compensate for the inevitable 10–20% loss to follow-up, the calculated sample size was set at 500 participants per group (Fig. 3).

**Fig. 4:**
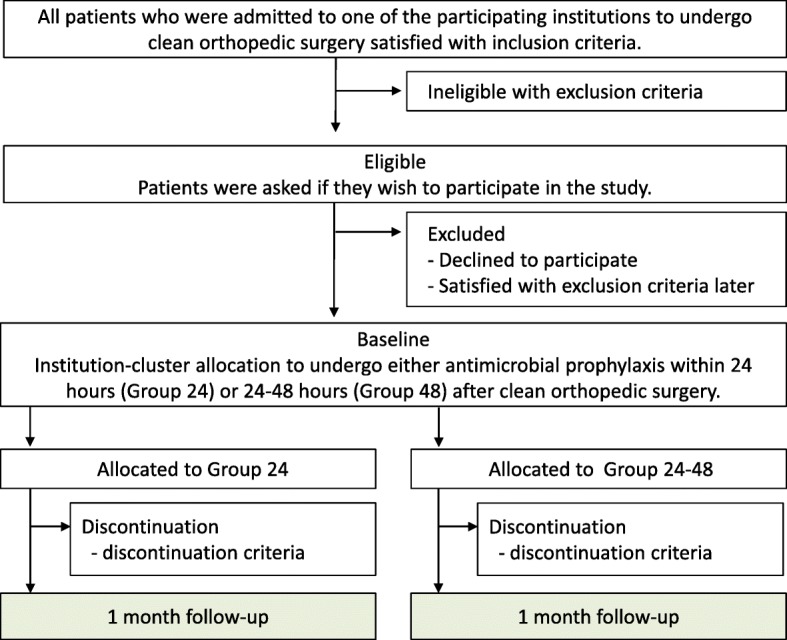
An overview of the time course. Health care-associated infections within 30 days after surgery will be evaluated between 30 and 180 days after surgery

### Inclusion and exclusion criteria

Inclusion criteria for the study are as follows: (1) age ≥ 20 years; (2) hospitalized patients; (3) patients who underwent clean surgery based on CDC wound classification system; (4) patients with a good command of the Japanese language; (5) patients able to give consent on their own or through relatives; and (6) a primary intention wound closure. Patients will be excluded from enrollment if they have undergone any of the following procedures: (1) operations involving the use of external fixations; (2) operations including amputations; (3) needle biopsies; (4) implant removal; (5) reconstructive surgery involving skin tissue, such as flap surgery; (6) antibiotic therapy prior to surgery; (7) percutaneous vertebroplasty (balloon kyphoplasty, vertebroplasty, etc.); and (8) procedures performed in collaboration with other departments.

The observation will be discontinued if patients meet any of the following criteria: (1) taking antibiotic, antiviral, antifungal, or antituberculosis drugs on the day of surgery; (2) operations deemed inappropriate for the study continuation due to adverse events or deterioration of complications or of the original disease, including anaphylaxis; (3) inclusion criteria were not met or any exclusion criterion applicable after registration; (4) discharge within 48 h after surgery; (5) patient refusal to continue participating in this study; and (6) continuation of participation deemed inappropriate by the participating doctor for other rational reasons. The participating doctors will be asked for a detailed description in this case.

### Trial design

In this study, patients will be divided into two groups of different AMP duration using the institution-by-period cluster allocation system. Patients will not be informed of allocation schedule preoperatively. In this predetermined cluster allocation, the participating hospitals will be divided into two protocols: protocol A and protocol B (Table 1). AMP administration will be discontinued within 24 h after wound closure in protocol A and within 24–48 h in protocol B. When a certain observation period (2–4 months) has elapsed, the administration period of protocol A will be changed to protocol B and vice versa. A pair of protocols A and B in a total of 4 months will be considered as one course. The course will be repeated until we achieve the planned number of participants (estimated at 8–12 months, considering the participating hospital records). Finally, we will evaluate the obtained data and compare patients who undergo either AMP within 24 h (Group 24) or within 24–48 h (Group 48). From our previous data, the patient backgrounds are expected to be similar between the two groups when conducted by the planned allocation method. Our hypothesis is that the SSI and HAI rate will be equivalent between Group 24 and Group 48.

**Table 1:** Cluster allocation

		May–June	July–August	Sept-Oct	Nov-Dec
Protocol A	Hospital A	Group 48	Group 24	Group 24	Group 48
Protocol B	Hospital B	Group 24	Group 48	Group 48	Group 24
Protocol A	Hospital C	Group 48	Group 24	Group 24	Group 48
Protocol B	Hospital D	Group 24	Group 48	Group 48	Group 24
Protocol A	Hospital E	Group 48	Group 24	Group 24	Group 48

### Intervention

All patients will receive an initial dose of 2 g of cefazolin before the initial incision, with an additional 1 g provided every 3–4 h during surgery, following the JSC/JSSI guideline (http://www.chemotherapy.or.jp/guideline/jyutsugo_shiyou_jissen.pdf) (Table 2). Vancomycin or clindamycin will be used for those with beta-lactam allergy. Preoperative administration of AMP was considered adequate if cefazolin was started within 60 min before surgical incision (120 min for vancomycin). When infectious diseases are suspected, and additional antibiotics are required, participating doctors will be free to give any antibiotic therapy necessary. They will be asked to report these events and to try their best to detect the source of infection and the causative pathogen. No restriction will be set on antimicrobial choice or duration for the purpose of SSI treatment. Spinal surgery without implants followed same protocol, as previous RCT showed that AMP was effective in this particular procedure [24]. Patients allocated to Group 24 will receive their final AMP intravenously within 24 h after surgery. Patients allocated to Group 48 will receive their AMP intravenously between 24 h and 48 h after closure. AMP duration will be measured starting from the time when the procedure is completed until when the final course of AMP is started.

**Table 2:** Administration methods of prophylactic antibiotics

Dose of the initial prophylactic antibiotics used preoperatively				
Cefazolin	2 g			
Vancomycin	1 - 1.5 g			
Clindamycin	600 mg			
Dose and interval of additional prophylactic antibiotics used intraoperatively				
		eGFR (ml/min)		
		>50	20−50	<20
Cefazolin	1 g	every 3 hours	every 8 hours	every 16 hours
Vancomycin	1 g	every 8 hours	every 16 hours	No recommendation
Clindamycin	600 mg	every 6 hours		

### Other SSI prevention measures

To minimize the effect of confounding factors, we have asked all the participating institutions to adhere closely to the following SSI prevention protocol:

1. Preoperative shaving by electrical clippers immediately before skin incision [25, 26], when necessary.
2. Preoperative skin preparation at least twice with povidone iodine with or without alcohol before using adhesive drapes [5, 27, 28].
3. All surgeons and operation staff to wear double gloves and change their outer gloves at least every 2 h during the operation [29, 30].
4. Iodine-impregnated adhesive drapes to be used after skin preparation in all procedures except for hand or foot surgeries [31].
5. Operation room traffic to be kept to a minimum.
6. A laminar air flow-equipped theater to be used for total arthroplasties [5, 32].
7. Initial AMP to be administered before surgery and completed before tourniquet inflation, with an additional dose during surgery based on renal function (Table 2) (http://www.chemotherapy.or.jp/guideline/jyutsugo_shiyou_jissen.pdf).
8. Forced-air warming systems to be used to maintain normothermia [5].
9. An antimicrobial-coated suture to be used for closure.
10. Local antibiotics, such as antibiotic-impregnated cement or vancomycin powder, to be avoided for SSI prevention at initial surgery.

Although, recent meta-analysis showed that the available evidence shows no benefit for laminar airflow compared with conventional turbulent ventilation in reducing the risk of SSIs [33], arthroplasties were routinely performed in laminar airflow theaters in participating hospitals. Considering the capacity of operating room theaters in each hospital, we have decided to follow our original practice.

### Follow-up duration and primary/secondary outcomes

The primary outcome will be the cumulative incidence of all postoperative bacterial infectious diseases which will be diagnosed within 30 days (the operation date being defined as “Day 0”) after surgery and requiring antibiotic therapy. Antiviral, antifungal, and anti-tuberculosis therapy will not be considered as therapies for postoperative bacterial infectious diseases unless antibiotics are used. Bacterial infectious diseases will be classified into (1) SSI, (2) UTI, (3) RTI, or (4) other infectious diseases. Secondary outcomes will include the prevalence of diseases (1)–(4), (5) mortality, (6) cardiovascular events observed within 30 days after surgery, (7) prolonged hospitalization for > 30 days, and (8) the rate of antibiotic resistance of SSI pathogens. In Japan, patients are usually hospitalized for more than 2 weeks following orthopaedic surgery. From our previous report, approximately 20% of patients were still hospitalized at day 30 after surgery [23]. We therefore decided to evaluate the rate of prolonged hospitalization for > 30 days, as well as shorter duration.

### Evaluation methods

All participating surgeons are responsible for data collection, and representatives from each participating institutions are responsible for data validation. Patient background, surgical information, and complications within 30 days after the surgeries will be recorded in our web database. These follow-up data will be recorded and evaluated within 30–180 days after the surgery (Fig. 4). The evaluation methods will be classified into four categories:

1. Direct evaluation by the surgeons in the operating institution.
2. Direct evaluation by the physicians outside the operating institution.
3. Evaluation by mail via questionnaires of the postoperative complications.
4. Evaluation by phone via questions on postoperative complications.

### Statistical method

Considering the non-inferiority hypothesis in the study, both intention-to-treat and per-protocol analyses will be performed as primary analyses. Intention-to-treat analysis will compare the groups defined according to the allocation, although AMP is not administered as allocated; we will include patients assigned to Group 48 but who underwent postoperative AMP administration within 24 h after surgery as Group 48, and vice versa*.* Per-protocol analysis will compare Group 24 with Group 48 after excluding patients who deviate from the allocation. We will exclude cases whose primary outcomes could not be recorded due to loss of follow-up assuming as missing will happen completely at random between the two groups.

The risks of the primary outcome will be compared between Groups 24 and 48 by the Farrington–Manning test for a 4% risk difference as a test hypothesis. We will not be adopting cluster randomization; rather, we will allocate patients to each intervention according to the predetermined “hospital-period cluster.” The 95% confidence intervals (CIs) of effect measures, such as risk difference and ratio, will be estimated through regression models (with and without adjusting for possible risk factors) fitted by generalized estimating equations (GEEs) that cluster hospital and periods, analogous to the cluster-randomized trial design.

For secondary outcomes, we will not test non-inferiority by setting non-inferiority margins; instead, we will evaluate whether there are excessive increases in these risks in Group 24 compared with Group 48 by estimating the 95% CI of risk differences and ratios through regression models (with and without adjusting for possible risk factors) fitted by the GEE approach that clusters hospital and periods.

Due to the short study period, an interim analysis will not be performed.

### Planned subgroup analyses

We will analyze the main and secondary endpoints in subgroups including patients’ background, surgical factors, for example, age, sex, diabetes mellitus, rheumatoid arthritis, hemodialysis, body weight, American Society of Anesthesiologists physical status, type of surgery, drainage use, and urinary catheter use. Continuous values will be divided into two groups with an appropriate cutoff. Multivariate analysis will be used.

### Informed consent and ethical issues

The study protocol was approved by the local ethics committees of all five participating hospitals. All participants have given documented informed consent before entry. The results will be disseminated in the academic forums, including peer-reviewed publications and presentations at domestic and international conferences. This study will be conducted in accordance with the Declaration of Helsinki.

### Adverse events

Adverse events, including anaphylaxis, will be assessed by the responsible surgeons and from chart reviews.

## Discussion

Adequate use of AMP is encouraged worldwide, but is generally discontinued as quickly as possible in consideration of the potential risk of antibiotic resistance when used too long [6]. Prolonged use of postoperative antibiotics is discouraged because of possible antimicrobial toxicity and unnecessary expenses [32]. In the recent WHO report, > 50% of *Staphylococcus aureus* was methicillin-resistance in several countries, including Japan and the US [34]. The threat of antimicrobial resistance is now a global concern, and the Japanese Ministry of Health, Labor and Welfare has launched a national action plan to reduce the methicillin-resistant *S. aureus* rate from 51 to < 20% by 2020, while reducing the total amount of antibiotics used (https://www.mhlw.go.jp/file/06-Seisakujouhou-10900000-Kenkoukyoku/0000120769.pdf). A shorter AMP duration may theoretically reduce the incidence of resistant pathogens as well as the total amount of antibiotics used. Therefore, shorter AMP is an attractive method to combat antimicrobial resistance and should be encouraged if its effectiveness for preventing postoperative complications is comparable to that of longer AMP duration.

Although there is a trend toward short AMP duration worldwide, several concerns require further investigations. First, shortening the duration of AMP may not prevent SSIs. The supportive evidence for preventing SSIs in clean surgery is still limited, and several studies indicated the possibility of a higher SSI risk when the AMP duration is too short [10, 15, 16]. Second, the short duration may consequently increase other HAIs postoperatively, but these relationships are not well established. Third, there is no high-level evidence study supports the notion that shorter AMP duration decreases antimicrobial resistance in the field of orthopaedic surgery. The evidence of higher resistance risk with longer AMP duration is mainly based on observational studies [35, 36], which may not be appropriate to generalize in various clean orthopaedic procedures.

Many Japanese orthopaedic surgeons still prefer AMP of longer durations longer than 24 h. From our previous study, about half the procedures performed in our institutions were continued for > 24 h. Surgeons in participating hospitals were not aware of the CDC/WHO recommendations at the time we planned this trial. Therefore, it was very difficult to conduct a trial comparing those cases without an additional dose after closure, as this was too short for most participating surgeons and was not part of our general practice. Although the JSC/JSSI guideline recommends AMP discontinuation within 48 h (http://www.chemotherapy.or.jp/guideline/jyutsugo_shiyou_jissen.pdf), [14], there is not enough evidence to justify prolonged AMP use for > 24 h in clean orthopaedic procedures. Unnecessary doses of antibiotics may only increase the chances of antimicrobial resistance as well as medical costs. Therefore, we have designed this comparative study to evaluate the efficacy of AMP duration, by comparing AMP duration of 24 h with that of 24–48 h after clean orthopaedic surgery.

Approximately 4% of patients in the US acute care hospitals have at least one HAI [22]. Postoperative HAIs are associated with greater deterioration in patients’ general health status and social/economic burden [6]. In this study, we have hypothesized that the risk of HAIs for 24 h of AMP will be equivalent to 24–48 h of AMP. If Group 24 reveals an equivalent or lower complication rate, this would encourage surgeons to shorten their AMP duration, which may minimize the chance of antibiotic resistance and reduce the antibiotic load. In contrast, if Group 24 reveals a higher HAI risk, we might have to reconsider longer AMP duration in regard to patient safety and push for future research to validate our results. Either result will be informative and has the potential to change our future practice. In Japan many surgeons still prefer AMP longer than 24 h. From the viewpoint of economics and potential resistance, shorter AMP duration should be encouraged. Therefore, we have chosen a non-inferiority design.

The limitation of this study design should be acknowledged. First, the allocation schedule which will be scheduled based on previous data to achieve similar background, will be announced to the participating hospitals before the study. This was to provide a reasonable time for preparing the study intervention, to avoid crossovers and decrease human errors regarding AMP administration. Thus, bias regarding allocation concealment may be present. To minimize the bias, participating hospitals were asked not to select patients based on our allocation schedules. We will also monitor the recruited number of patients, and explore patient backgrounds of the study populations thoroughly to assure comparability. Though patients may recognize which group they belong after 24 h after surgery, they do not know which group they belong at the time when they receive surgery. Bias regarding patients’ preference is less likely to be present, and it is hardly imaginable that the outcome may be influenced by unblinded patients. Second, the variation of doses repeated within and after 24 h in both groups may influence the results. Though, we have strictly controlled not to extend the planned duration for Group 24, and not to forget additional dose after 24 h for Group 48, to avoid cross overs and to improve protocol adherence, the number and amount of doses repeated in both groups will not be strictly controlled considering wide variety of procedures that are included in this study, and to maintain feasibility of our participating hospitals.

The NOCOTA study has been designed to provide evidence to determine adequate AMP duration, not just to prevent SSIs, but to prevent overall infectious postoperative complications. We believe that the results of this trial will have a substantial impact on the future management of clean orthopaedic surgery and provide deeper insights into patient safety.

## Trial status

The trial was registered in the UMIN000030929 and opened on January 22, 2018. The first patient was assigned on May 1, 2018. Patient recruitment was planned to be open between May 1, 2018 and December 31, 2018.

## Data Availability

The datasets used and/or analyzed during the current study are available from the corresponding author on reasonable request.

## Abbreviations

AMP: Antimicrobial prophylaxis
ASHP: American Society of Health-System Pharmacists
CDC: Center for Disease Control and Prevention
CI: Confidence interval
GEE: Generalized estimating equations
GRADE: Grading of Recommendations Assessment, Development and Evaluation
JSC: Japanese Society of Chemotherapy
JSSI: Japan Society for Surgical Infection
MSIS: Musculoskeletal Infection Society
RCT: Randomized control trials
RTI: Respiratory tract infection
SSI: Surgical site infections
UTI: Urinary tract infection
WHO: World Health Organization
